# The impact of temperature on insecticide toxicity against the malaria vectors *Anopheles arabiensis* and *Anopheles funestus*

**DOI:** 10.1186/s12936-018-2250-4

**Published:** 2018-04-02

**Authors:** Katey D. Glunt, Shüné V. Oliver, Richard H. Hunt, Krijn P. Paaijmans

**Affiliations:** 1 ISGlobal, Barcelona Ctr. Int. Health Res. (CRESIB), Hospital Clínic - Universitat de Barcelona, Barcelona, Spain; 2 Centre for Emerging Zoonotic and Parasitic Diseases, National Institute for Communicable Diseases, Johannesburg, South Africa; 3 Wits Research Institute for Malaria, School of Pathology, Faculty of Health Sciences, University of the Witwatersrand, Johannesburg, South Africa; 4 Centro de Investigação em Saúde de Manhiça (CISM), Maputo, Mozambique

**Keywords:** *Anopheles funestus*, *Anopheles arabiensis*, WHO tube bioassays, Environmental variation, Insecticide resistance, Malaria elimination, Vector control, Temperature

## Abstract

**Background:**

It is anticipated that malaria elimination efforts in Africa will be hampered by increasing resistance to the limited arsenal of insecticides approved for use in public health. However, insecticide susceptibility status of vector populations evaluated under standard insectary test conditions can give a false picture of the threat, as the thermal environment in which the insect and insecticide interact plays a significant role in insecticide toxicity.

**Methods:**

The effect of temperature on the expression of the standard WHO insecticide resistance phenotype was examined using *Anopheles arabiensis* and *Anopheles funestus* strains: a susceptible strain and the derived resistant strain, selected in the laboratory for resistance to DDT or pyrethroids. The susceptibility of mosquitoes to the pyrethroid deltamethrin or the carbamate bendiocarb was assessed at 18, 25 or 30 °C. The ability of the pyrethroid synergist piperonyl-butoxide (PBO) to restore pyrethroid susceptibility was also assessed at these temperatures.

**Results:**

Temperature impacted the toxicity of deltamethrin and bendiocarb. Although the resistant *An. funestus* strain was uniformly resistant to deltamethrin across temperatures, increasing temperature increased the resistance of the susceptible *An. arabiensis* strain. Against susceptible *An. funestus* and resistant *An. arabiensis* females, deltamethrin exposure at temperatures both lower and higher than standard insectary conditions increased mortality. PBO exposure completely restored deltamethrin susceptibility at all temperatures. Bendiocarb displayed a consistently positive temperature coefficient against both susceptible and resistant *An. funestus* strains, with survival increasing as temperature increased.

**Conclusions:**

Environmental temperature has a marked effect on the efficacy of insecticides used in public health against important African malaria vectors. Caution must be exercised when drawing conclusions about a chemical’s efficacy from laboratory assays performed at only one temperature, as phenotypic resistance can vary significantly even over a temperature range that could be experienced by mosquitoes in the field during a single day. Similarly, it might be inappropriate to assume equal efficacy of a control tool over a geographic area where local conditions vary drastically. Additional studies into the effects of temperature on the efficacy of insecticide-based interventions under field conditions are warranted.

**Electronic supplementary material:**

The online version of this article (10.1186/s12936-018-2250-4) contains supplementary material, which is available to authorized users.

## Background

The development and spread of insecticide resistance in mosquito vectors is thought to be a major threat for malaria control and elimination programmes worldwide. Resistance to pyrethroid insecticides has been identified in populations of malaria vectors across Africa [1, 2] and, given the central role of this class of insecticides in insecticide-based vector control (the only one approved by the WHO to be used in treated bed nets and the most-used in indoor residual sprays [3]) the increasing prevalence of resistance is regarded with concern, as it may undermine malaria control and elimination activities [4, 5].

Continuous monitoring of insecticide susceptibility in malaria vector populations informs the choice of chemicals to be used in an area and allows for the management of insecticide resistance [6]. However, insecticide toxicity does not only depend on the active ingredient. The efficacy of a chemical against its target is also a function of the formulation, the biology of the insect, and the environment in which these interact [7]. Thus, it is difficult to predict how an insecticide susceptibility test in a laboratory or insectary, where insecticide dose, mosquito physiological status (e.g., age, blood feeding, larval nutrition) and climate are controlled [8], translates to the efficacy of an insecticide in the field [9].

Environmental temperature in particular has been shown to influence the outcome of insecticide exposure; temperature differences expected to occur naturally under field conditions can lead to notable variations in chemical efficacy [10]. The importance of such changes in effectiveness to the control of malaria vectors has not been widely considered, though temperature-dependent sensitivity to insecticides has been demonstrated in *Anopheles gambiae* (pyrethroid permethrin: [11], pyrrole chlorfenapyr: [12]) and *Anopheles stephensi* with varying levels of resistance (organochlorine DDT and organophosphate diazinon: [13], permethrin: [11], permethrin and organophosphate malathion: [14]).

Here, the effects of temperature on the expression of insecticide resistance in *Anopheles arabiensis* and *Anopheles funestus* were examined by exposing susceptible and resistant strains of these species to the pyrethroid deltamethrin or the carbamate bendiocarb at 18, 25 or 30°C. The ability of the pyrethroid synergist piperonyl butoxide (PBO) to restore susceptibility in pyrethroid-resistant mosquitoes was also evaluated at these temperatures. This is the first investigation into the effects of temperature on insecticide susceptibility in these major vectors of southern Africa, and the first look at the contribution of environmental temperature to the efficacy of PBO in resistant mosquitoes.

## Methods

### Mosquito strains

For each of two *Anopheles* species, two strains of mosquitoes were used: a parent strain and a strain derived from the parent by insecticide selection (Table 1). All experiments were carried out in the Vector Control Research Laboratory (VCRL) in Johannesburg, South Africa. Strains were reared and maintained according to their standard procedures, described in Hunt et al. [15].

**Table 1:** Species tested and their susceptibility status

Species	Colony name	Resistance
*An. arabiensis*	SENN	Low level to permethrin
	SENN-DDT	DDT, permethrin, deltamethrin, and malathion
*An. funestus*	FUMOZ	Pyrethroids and bendiocarb
	FUMOZ-R	Pyrethroids and bendiocarb

SENN is an *An. arabiensis* strain from Sennar, Sudan, that has been maintained at the VCRL since 1990. As described in Oliver and Brooke [16], SENN-DDT was selected by exposing 1–3 days old non-blood fed SENN mosquitoes to 4% DDT for 1 h and then allowing survivors to breed. This procedure, repeated each generation since 1995, has selected for multiple insecticide resistance (DDT, permethrin, deltamethrin, malathion), mediated by increased detoxification enzyme activity and fixation of the L1014F *kdr* mutation [16, 17].

FUMOZ is an *An. funestus* strain from southern Mozambique that has been maintained at the VCRL since 2000. Similar to the method used to generate the SENN-DDT strain, selection pressure with 0.75% permethrin, a pyrethroid, was used to generate the FUMOZ-R strain. Although selection ceased in 2005, both strains remain resistant to pyrethroids and carbamates. No *kdr* alleles are present in either *An. funestus* strain [18]. The mechanism of resistance in these mosquitoes is metabolic in nature [19, 20]. Updated assessments of the resistance status of SENN-DDT, FUMOZ and FUMOZ-R are described in Venter et al. [21].

### Temperature treatment and insecticide exposure

Exposures to 0.05% deltamethrin and 0.1% bendiocarb followed the WHO insecticide-resistance monitoring ‘tube test’ protocol [8], using test papers acquired from the WHO. To evaluate the effect of temperature on insecticide susceptibility, two temperature treatments were included in addition to the standard 25 °C specified in the tube test protocol. The low temperature treatment, 18 °C, represents a possible average night-time temperature or the average daily African highland temperature, while the high temperature treatment, 30 °C, is a temperature that might be expected closer to mid-day, or an average in some parts of sub-Saharan Africa during the summer [22]. Incubators (0.32 m × 0.31 m × 0.33 m) and thermoregulators (Sable Systems, North Las Vegas, NV, USA) were used to maintain conditions at 18 and 30 °C; wet towels were added to maintain relative humidity (RH) over 70%. The VCRL insectary provided the standard environment (~ 25 °C, 80% RH). Temperature and humidity were monitored at 5-min intervals with USB data loggers (SSN-22, AWR Smith Process Instrumentation, South Africa). Mean and standard deviation of temperature and per cent RH for each experiment are listed in the supplementary information (Additional file 1: Table S1), and the averages for all experiments are listed in Table 2.

**Table 2:** Sample sizes and mean environmental conditions

*Anopheles* spp.	Strain	Insecticide	# Exp. replicates	Treatment (°C)	n (# of females)		Temperature (°C)		RH (%)	
					Control	Insecticide	Mean ± SD	(Min, Max)	Mean ± SD	(Min, Max)
*arabiensis*	SENN	Deltamethrin	2	18	99	199	18.25 ± 0.24	(18, 22.5)	89.93 ± 7	(54.9, 97.1)
				25	96	188	25.79 ± 1.67	(24.3, 29)	79.27 ± 5.98	(58.3, 88.5)
				30	99	191	30.24 ± 0.42	(27.5, 32.1)	91.25 ± 5.38	(40.2, 96.9)
	SENN-DDT	Deltamethrin	5	18	216	474	18.35 ± 0.54	(13.5, 21.2)	82.44 ± 13.99	(40.5, 98.4)
				25	203	501	25.47 ± 1.17	(22.4, 28.1)	79.99 ± 4.06	(64.3, 91.7)
				30	211	495	30.16 ± 0.50	(28.6, 31.1)	88.03 ± 8.63	(37.1, 99.8)
		+PBO^a^	2	18	46	203	18.95 ± 0.84	(13.4, 21.2)	60.68 ± 7.57	(40.5, 95.1)
				25	53	198	25.17 ± 1.51	(22.4, 27.6)	79.30 ± 4.24	(68.2, 88.4)
				30	55	198	30.38 ± 0.12	(30.0, 30.7)	84.83 ± 9.23	(39.5, 98.7)
*funestus*	FUMOZ	Deltamethrin	3	18	121	318	18.55 ± 1.02	(14.6, 25.1)	90.36 ± 11.66	(33.9, 99.7)
				25	132	301	25.24 ± 0.74	(23.9, 30.9)	80.98 ± 4.03	(26.1, 85.4)
				30	128	306	30.60 ± 0.25	(29.9, 31.2)	85.95 ± 15.65	(27.2, 99.0)
		+PBO^a^	1	*18*	28	101	18.70 ± 0.03	(18.7, 18.8)	98.78 ± 0.88	(95.4, 99.7)
				25	27	107	25.68 ± 0.43	(24.6, 26.8)	81.00 ± 1.04	(77.8, 83.0)
				30	26	98	30.49 ± 0.11	(30.3, 30.8)	77.23 ± 2.41	(65.7, 81.9)
	FUMOZ-R	Deltamethrin	2	18	88	215	18.37 ± 0.90	(14.4, 19.0)	92.77 ± 6.29	(65.6, 100)
				25	70	216	25.05 ± 1.08	(21.8, 26.9)	83.57 ± 4.10	(74.5, 91.7)
				30	86	222	30.07 ± 0.43	(29.5, 30.8)	90.97 ± 9.50	(57.5, 100)
		+PBO^a^	1	18	26	101	18.74 ± 0.24	(17.2, 19.0)	97.73 ± 2.87	(81, 100)
				25	28	104	24.44 ± 0.91	(21.8, 26.3)	79.93 ± 1.83	(74.5, 83.0)
				30	23	100	30.49 ± 0.16	(30.0, 30.8)	81.90 ± 4.78	(57.5, 88.9)
	FUMOZ	Bendiocarb	2	18	89	195	18.36 ± 0.07	(18.3, 21.8)	93.87 ± 2.05	(69.4, 97.3)
				25	101	196	24.89 ± 0.44	(23.6, 26.6)	88.49 ± 3.78	(74, 96.8)
				30	105	195	29.09 ± 0.14	(26.9, 30.6)	86.35 ± 4.4	(41.8, 92.5)
	FUMOZ-R		3	18	139	310	18.42 ± 0.19	(18.3, 22.4)	90.08 ± 3.47	(55.2, 94.3)
				25	147	292	25.18 ± 0.84	(23.4, 28.3)	84.28 ± 3.85	(66.3, 98.3)
				30	133	293	30.21 ± 0.11	(29.3, 30.9)	89.58 ± 4.25	(58.2, 98.4)

^a^In experiments that included PBO treatments, the “Control” column is the positive control, or the mosquitoes exposed to PBO-only, and the “Insecticide” column are those mosquitoes exposed to deltamethrin + PBO

For each temperature, groups of approximately 25 female mosquitoes were transferred by mouth aspirator to plain paper-lined holding tubes: two tubes were designated as control replicates (therefore, ~ 50 control females), 4 as insecticide-exposed replicates (~ 100 exposed females). Tubes were moved to their respective temperature treatments, and mosquitoes were given 1 h to acclimate before the 1-h exposure. Acclimatization and exposure occurred during mosquito scotophase (‘mosquito night’). At the end of the exposure to control or insecticide-treated papers, mosquitoes were moved back to the holding tubes and sucrose was provided. Mosquitoes were kept at their treatment temperatures for 24 h in the dark (due to equipment limitations), after which mortality was recorded. Experiments with each mosquito strain were replicated to generate the sample sizes in Table 2. Number of experimental replicates: using deltamethrin SENN: 2, SENN-DDT: 5; FUMOZ: 3; FUMOZ-R: 2; using bendiocarb FUMOZ: 2; FUMOZ-R: 3.

The effect of the pyrethroid synergist piperonyl-butoxide (PBO) on the toxicity of deltamethrin at these temperatures was also assessed. At each temperature, there were 4 control or insecticide treatments: untreated control, PBO-only control, deltamethrin-only, deltamethrin + PBO. PBO-only and deltamethrin + PBO groups were exposed to papers treated with 4% PBO [23] post-acclimatization, for 30 min prior to the 1-h experimental period. Thirty minutes was used because a 1-h exposure to PBO alone at 30°C induced 100% mortality. Number of experimental replicates: SENN: not tested, SENN-DDT: 2; FUMOZ: 1; FUMOZ-R: 1.

### Data analysis

Statistical analyses were performed in R v. 3.2.1 [24]. Mortality data (number of mosquitoes dead and alive per tube) were analysed with generalized linear models, using a binomial error distribution and logit link function, to assess the affect of treatment on mosquito survival. Temperature was the independent variable (low, standard, high), and was coded as a categorical variable, with the low group as the reference level. Experimental replicate was included as a random effect. Groups with consistently ~ 0 or ~ 100% mortality (e.g., control or deltamethrin + PBO groups) could not be included in analysis due to complete separation.

## Results

### *Anopheles arabiensis* strains

**Fig. 1:**
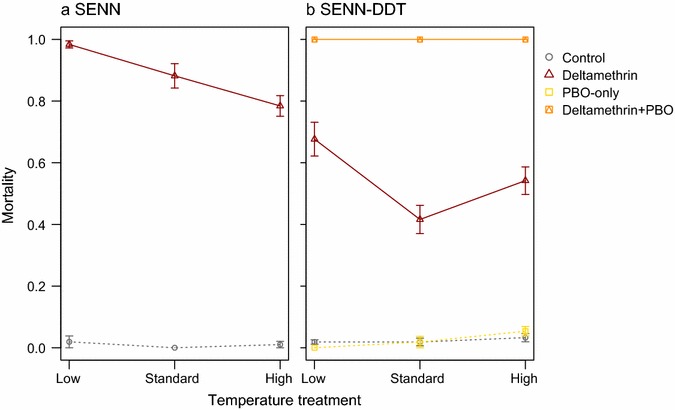
Temperature affects the survival of female *Anopheles arabiensis* exposed to deltamethrin. **a** In the unselected *An. arabiensis* strain, SENN, deltamethrin displayed a consistently negative temperature coefficient, its toxicity decreasing with increasing temperature. **b** SENN-DDT females, however, are more likely to survive deltamethrin exposure under standard insectary conditions; the probability of dying increased at both the lower and higher temperatures. PBO pre-exposure completely restored susceptibility to deltamethrin

Only insecticide-exposed mosquitoes were included in the analysis of SENN experiments due to uniformly high control survival across temperatures. Similarly, control mosquitoes were excluded from the SENN-DDT analysis, as their survival was not affected by temperature (Fig. 1b; Χ^2^ = 1.1, df = 2, p = 0.6).

Temperature significantly influenced the probability of unselected and selected *An. arabiensis* strains dying from deltamethrin exposure (Fig. 1; SENN: χ^2^ = 30.3, df = 2, p < 0.001; SENN-DDT: χ^2^ = 17.2, df = 2, p < 0.001). However, the changes in susceptibility with temperature differed slightly between the two strains. Deltamethrin toxicity in the unselected SENN strain consistently decreased as temperature increased; thus, deltamethrin displayed what is called a negative temperature coefficient. SENN had significantly greater odds of dying at the low temperature, compared to the standard 25 °C (Table 3). Deltamethrin was less toxic to SENN-DDT females at the standard temperature than at either extreme; both the low and high temperatures increased the likelihood of being killed by deltamethrin exposure (Fig. 1b, Table 3). PBO exposure completely restored susceptibility to deltamethrin in SENN-DDT at all temperatures (Fig. 1b).

**Table 3:** Odds of mosquitoes at a given temperature treatment dying following deltamethrin exposure

Species	Strain	Temperature treatment	Odds ratio	95% CI
*An. arabiensis*	SENN	Low	76.5	18.0, 325.0
		Standard	0.1	0.03, 0.4
		High	0.1	0.02, 0.2
	SENN-DDT	Low	2.2	1.2, 4.2
		Standard	0.3	0.2, 0.4
		High	0.5	0.4, 0.7
*An. funestus*	FUMOZ	Low	1.5	0.9, 2.5
		Standard	0.3	0.2, 0.4
		High	1.6	1.1, 2.2

### *Anopheles funestus* strains

**Fig. 2:**
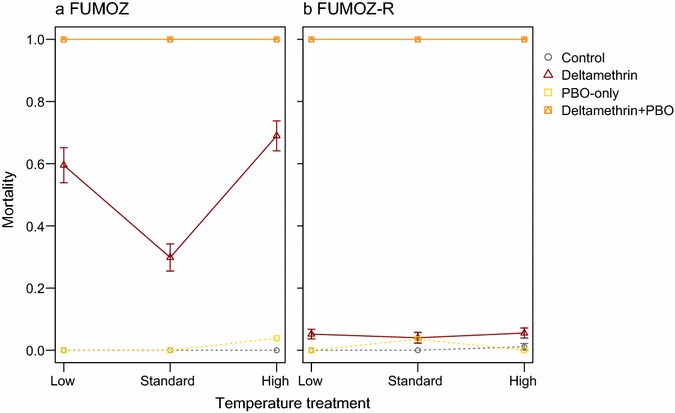
Effects of temperature on *Anopheles funestus* susceptibility to deltamethrin depends on resistance levels. **a** In the unselected *An. funestus* strain, FUMOZ, individuals were most likely to survive deltamethrin exposure under standard insectary conditions; both higher and lower temperatures increased the efficacy of deltamethrin. **b** Temperature did not have a marked effect on deltamethrin-induced morality in the more resistant, selected FUMOZ-R strain. In both strains, PBO pre-exposure completely restored susceptibility to deltamethrin

**Fig. 3:**
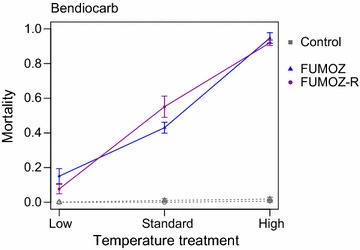
Temperature affects the survival of female *Anopheles funestus* mosquitoes exposed to bendiocarb. Bendiocarb displayed a strongly positive temperature coefficient in both unselected and selected strains, becoming more toxic with increasing temperature

Due to uniformly high survival in control groups of both *An. funestus* strains, and uniformly high mortality in deltamethrin + PBO-exposed groups (Figs. 2, 3, dotted lines), only mosquitoes exposed to deltamethrin- or bendiocarb-only were included in the analyses.

Although survival to deltamethrin in the selected FUMOZ-R was consistently high across temperatures (Fig. 2b; FUMOZ-R: χ^2^ = 0.8, df = 2, p = 0.7), temperature significantly influenced the probability of the unselected FUMOZ females being killed by exposure to the pyrethroid deltamethrin (Fig. 2a; FUMOZ: χ^2^ = 111.7, df = 2, p < 0.001). FUMOZ exposed at 18° or 30 °C had a greater risk of dying than those exposed at the WHO standard exposure temperature (Table 3). PBO exposure completely restored susceptibility to deltamethrin in both of these *An. funestus* colonies, at all temperatures (Fig. 2).

In both *An. funestus* strains, temperature significantly affected the probability of surviving exposure to bendiocarb (Fig. 3; FUMOZ: χ^2^ = 49.0, df = 2, p < 0.001; FUMOZ-R: χ^2^ = 66.7, df = 2, p < 0.001). Bendiocarb displayed a consistently positive temperature coefficient, with mortality increasing as temperature increased.

## Discussion

Here it was demonstrated for the first time that: (1) temperature impacted insecticide toxicity in *An. arabiensis* and *An. funestus*; (2) temperature affected the toxicity of deltamethrin and bendiocarb differently; and, (3) the synergist PBO fully restored pyrethroid susceptibility independent of temperature. These chemicals are of interest due to their utility in current (i.e., pyrethroids [3, 4]), and future (i.e., synergist PBO, incorporated into the next generation of pyrethroid-LLINs [25]) vector control interventions.

Temperature has long been known to be a critical factor underlying insecticide toxicity [7, 9, 11], and has now been demonstrated to impact the efficacy of public health insecticides against a number of malaria vector species (*An. gambiae* and *An. stephensi* [11, 12, 14]; *An. arabiensis* and *An. funestus*, this paper). The relationship between insecticide-induced toxicity and temperature is described in terms of a temperature coefficient (TC). If toxicity increases as temperature increases, the chemical has a positive TC. In other insects, DDT and pyrethroid insecticides often have negative TCs, i.e., toxicity decreases with increasing temperature, so the chemical is most effective at lower temperatures [7]. Here, the effect of temperature on deltamethrin toxicity depended on mosquito strain and its temperature coefficient was not always consistently positive or negative. Against both FUMOZ and SENN-DDT females (two different species and selection backgrounds, but similar levels of deltamethrin tolerance/resistance) this pyrethroid was more lethal at temperatures both lower and higher than the standard insectary temperature. Hodjati and Curtis also observed a non-linear (bi-modal) change in pyrethroid toxicity with temperature in *An. stephensi* exposed to permethrin [11]. They discussed the potential interaction of chemical toxicity or nerve sensitivity and mosquito behaviour over different temperatures. Although behaviour could not be observed in these experiments, the influence of temperature on mosquito activity levels is likely to be especially important in the case of pyrethroid insecticides, which are characterized by their effects on mosquito behaviour: irritancy and knockdown [7]. As the L1014F kdr mutation is present only in the SENN-DDT strain [21], a difference in neural sensitivity may underpin some of the variation seen between the strains.

Interestingly, the ability of the synergist PBO to restore pyrethroid susceptibility was not affected by temperature. At the temperatures tested, all mosquitoes exposed to PBO and then deltamethrin died. This is the first examination of the interaction between PBO and temperature in mosquitoes, and is especially important given that the next generation of long-lasting insecticidal nets (LLINs) that have received interim approval from the WHO to integrate PBO as a pyrethroid resistance countermeasure [25, 26]. Although it is unclear how the addition of PBO might drive increases in the intensity of resistance in mosquito populations, these experiments indicate that PBO could be effective in restoring susceptibility to deltamethrin independent of climatic conditions (in comparison to other novel vector interventions [12]). One possibility is that the addition of PBO as a selection pressure may shift the mechanism of pyrethroid resistance from P450-based to an alternative mechanism as, although P450s are the most common metabolic detoxification mechanism, it is not the only one [27]. Another important observation was that PBO alone for 1 h killed mosquitoes at 30 °C. The increased toxicity of PBO at high temperatures may be a manifestation of an increased reaction rate of irreversible inhibition at the higher temperature; given time, PBO will shift from being a synergist (defined in part by its lack of insecticidal activity on its own) to being toxic [28].

It is important to keep in mind that the outcomes presented here relate to the standard WHO susceptibility tests [8]. However, the observed drastic change in susceptibility over the three temperatures (that are reasonable in African transmission settings [22]), along with the increasing need to integrate non-pyrethroids into control programmes, reinforces the need for further investigation into the influence of temperature on insecticide toxicity. As demonstrated here (and elsewhere [10, 14]), it is important to consider local environmental conditions when monitoring insecticide resistance, as they have the potential to alter the outcomes and thus to affect conclusions and actions needed [9]. As such this work contributes to the ongoing debate on the limitations of the WHO tube assay [29, 30]. For example, SENN would be classified as resistant to deltamethrin, but exposures at cooler temperatures kill enough mosquitoes for SENN to be classified as susceptible. In addition, FUMOZ and FUMOZ-R are resistant to bendiocarb at the standard test temperature. However, when exposed to warmer temperatures mortality increases to 95 and 92%. Although both would still be classified as ‘resistant’, as mortality is below the WHO threshold of 98%, these differences (approx. onefold increase in mortality) may have a significant epidemiological impact. As the current and future chemical arsenal is limited [31] and resistance to multiple insecticide classes is now common [3], an adequate qualification of resistance is critical to maximize the number of available effective tools in the vector control toolbox.

Perhaps more importantly, this phenomenon (observed temperature-toxicity effects) may apply to actual chemical vector control interventions (LLINs, IRS or other chemical-based interventions, such as durable wall liners). Tools may be more or less effective under certain conditions, given that there are strong effects of temperature on toxicity. This was recently highlighted for chlorfenapyr, a pyrrole insecticide being evaluated for inclusion on LLINs for pyrethroid-resistance management [32]. Chlorfenapyr displayed a strongly positive temperature coefficient between 21 and 29 °C against the susceptible KISUMU strain of *An. gambiae*: while 82–100% of mosquitoes were killed at 27 °C, exposure at 22 °C killed 12–45% [12]. Although it is unclear what this difference means in terms of loss of disease control, it is clear that the importance of temperature in determining the efficacy of tools (and of other well-known factors, such as mosquito age, blood-feeding status, available dose, circadian rhythm [see 9]) adds to the complexity of the ongoing debate on the impact of insecticide resistance on intervention efficacy and transmission intensity [1, 33–36].

## Conclusion

Given their utility, insecticide-based vector control tools will continue to play a crucial role in malaria control and elimination strategies [37]. As novel active ingredients for public health insecticides are expected to be delivered no earlier than 2020 [1], and the second generation of non-pyrethroid, multi-insecticide-treated nets will only be available for widescale deployment in several years’ time, the current chemical arsenal needs to be deployed to its maximum potential, which may require us to think beyond laboratory insecticide resistance results. Performing efficacy tests with actual vector control products and local (wild-caught) vectors under real, field conditions (which would include exposures during the appropriate season and relevant time of day) would limit the impact of confounding factors (such as temperature, shown in this paper) and yield more accurate entomological intelligence for evidence-based decision-making.

## Additional file

**Additional file 1: Table S1.** Sample sizes and mean environmental conditions for each individual test.
