# Supplementary material for: The impact of temperature on insecticide toxicity against the malaria vectors Anopheles arabiensis and Anopheles funestus
Source: Malar J. 2018 Apr 2;17:131. doi: 10.1186/s12936-018-2250-4 (PMC5879579; doi:10.1186/s12936-018-2250-4)
Supplement: Supplementary file 1 — Additional file 1: Table S1. Sample sizes and mean environmental conditions for each individual test. [file 12936_2018_2250_MOESM1_ESM.docx]

**Table S1. Sample sizes and mean environmental conditions for each individual test**

|  |  |  |  |  | **n (# of females)** | | **Temperature (°C)** | | | | **RH (%)** | | | |
| --- | --- | --- | --- | --- | --- | --- | --- | --- | --- | --- | --- | --- | --- | --- |
| *Anopheles spp.* | Insecticide | Strain | Experimental replicate | Treatment | Control | Insecticide | Min | Max | Mean | SD | Min | Max | Mean | SD |
| *arabiensis* | Deltamethrin | SENN | 1(29Nov) | **18** | 45 | 95 | 18 | 22.5 | 18.16 | 0.37 | 63.4 | 96.6 | 92.08 | 3.79 |
|  |  |  |  | **25** | 48 | 88 | 24.3 | 29 | 26.58 | 1.67 | 58.3 | 88.1 | 76.41 | 5.98 |
|  |  |  |  | **30** | 44 | 89 | 27.5 | 32.1 | 30.11 | 0.42 | 53.7 | 96.9 | 92.41 | 5.38 |
|  |  |  | 2(31Aug) | **18** | 54 | 104 | 18.2 | 18.9 | 18.33 | 0.11 | 54.9 | 97.1 | 87.78 | 10.20 |
|  |  |  |  | **25** | 48 | 100 | 24.5 | 26.0 | 25.00 | 0.29 | 68.7 | 88.5 | 82.12 | 3.18 |
|  |  |  |  | **30** | 55 | 102 | 30.2 | 30.8 | 30.36 | 0.14 | 40.2 | 94.7 | 90.09 | 4.82 |
|  |  | SENN-DDT | 1 (2Dec) | **18** | 51 | 77 | 18.1 | 20.4 | 18.13 | 0.19 | 58.3 | 92.7 | 89.34 | 6.07 |
|  |  |  |  | **25** | 53 | 98 | 24.4 | 28.1 | 26.03 | 1.14 | 64.3 | 91.7 | 81.85 | 4.74 |
|  |  |  |  | **30** | 51 | 99 | 28.7 | 29.6 | 29.50 | 0.06 | 39.9 | 79.2 | 75.90 | 7.36 |
|  |  |  | 2 (9Dec) | **18** | 53 | 100 | 18.2 | 20.4 | 18.23 | 0.19 | 61.2 | 91.3 | 86.02 | 6.06 |
|  |  |  |  | **25** | 50 | 94 | 24.3 | 28 | 26.29 | 1.06 | 64.7 | 88.6 | 78.81 | 5.68 |
|  |  |  |  | **30** | 53 | 100 | 28.6 | 29.6 | 29.33 | 0.08 | 53.9 | 99.8 | 94.97 | 5.88 |
|  |  |  | 3 (25Aug) | **18** | 56 | 101 | 18.0 | 18.9 | 18.16 | 0.11 | 56.0 | 98.4 | 89.73 | 7.21 |
|  |  |  |  | **25** | 51 | 105 | 23.7 | 26.7 | 25.25 | 0.88 | 70.7 | 86.2 | 80.13 | 3.13 |
|  |  |  |  | **30** | 57 | 97 | 29.9 | 31.3 | 30.40 | 0.36 | 37.1 | 96.4 | 90.52 | 5.44 |
|  |  |  | 4 (17Jun)  +PBO* | **18** | 27 | 102 | 13.5 | 21.2 | 19.16 | 1.11 | 57.5 | 95.1 | 65.33 | 4.63 |
|  |  |  |  | **18** | 22 | 99 |  |  |  |  |  |  |  |  |
|  |  |  |  | **25** | 21 | 98 | 22.7 | 27.6 | 25.31 | 1.62 | 68.2 | 88.4 | 78.87 | 5.2 |
|  |  |  |  | **25** | 27 | 98 |  |  |  |  |  |  |  |  |
|  |  |  |  | **30** | 28 | 97 | 30.0 | 30.8 | 30.42 | 0.11 | 40.1 | 90 | 79.43 | 5.58 |
|  |  |  |  | **30** | 29 | 100 |  |  |  |  |  |  |  |  |
|  |  |  | 5 (12Aug)  +PBO* | **18** | 29 | 94 | 17.3 | 19.2 | 18.74 | 0.32 | 40.5 | 83.5 | 56.44 | 7.37 |
|  |  |  |  | **18** | 24 | 104 |  |  |  |  |  |  |  |  |
|  |  |  |  | **25** | 28 | 106 | 22.4 | 27.4 | 25.02 | 1.37 | 73.4 | 85.0 | 79.74 | 2.88 |
|  |  |  |  | **25** | 26 | 100 |  |  |  |  |  |  |  |  |
|  |  |  |  | **30** | 22 | 102 | 30.0 | 30.6 | 30.34 | 0.11 | 39.5 | 98.7 | 90.48 | 9.09 |
|  |  |  |  | **30** | 26 | 95 |  |  |  |  |  |  |  |  |

|  |  |  |  |  | **n (# of females)** | | **Temperature (°C)** | | | | **RH (%)** | | | |
| --- | --- | --- | --- | --- | --- | --- | --- | --- | --- | --- | --- | --- | --- | --- |
| *Anopheles spp.* | Insecticide | Strain | Experimental replicate | Treatment | Control | Insecticide | Min | Max | Mean | SD | Min | Max | Mean | SD |
| *funestus* | Deltamethrin | FUMOZ | 1(20Aug)^+^ | **18** | 51 | 100 | 14.5 | 21.2 | 19.43 | 1.38 | 54.5 | 99.6 | 93.35 | 8.53 |
|  |  |  |  | **25** | 52 | 96 | 23.9 | 26.6 | 25.21 | 0.81 | 67.5 | 84.1 | 79.69 | 3.53 |
|  |  |  |  | **30** | 50 | 95 | 28.5 | 31.2 | 30.47 | 0.36 | 34.5 | 99.0 | 90.06 | 14.32 |
|  |  |  | 2 (27Aug) | **18** | 48 | 110 | 18.0 | 25.1 | 18.34 | 0.94 | 33.9 | 98.7 | 88.14 | 12.38 |
|  |  |  |  | **25** | 52 | 105 | 23.9 | 26.6 | 25.13 | 0.69 | 38.8 | 85.4 | 81.2 | 3.04 |
|  |  |  |  | **30** | 52 | 105 | 30.1 | 31.2 | 30.65 | 0.22 | 31.2 | 98.5 | 86.96 | 16.67 |
|  |  |  | 3 (22Feb)  +PBO* | **18** | 22 | 108 | 18.7 | 18.8 | 18.70 | 0.03 | 95.4 | 99.7 | 98.78 | 0.88 |
|  |  |  |  | **18** | 28 | 101 |  |  |  |  |  |  |  |  |
|  |  |  |  | **25** | 28 | 100 | 24.6 | 26.8 | 25.68 | 0.43 | 77.8 | 83.0 | 81.00 | 1.04 |
|  |  |  |  | **25** | 27 | 107 |  |  |  |  |  |  |  |  |
|  |  |  |  | **30** | 26 | 106 | 30.3 | 30.8 | 30.49 | 0.11 | 65.7 | 81.9 | 77.23 | 2.41 |
|  |  |  |  | **30** | 26 | 98 |  |  |  |  |  |  |  |  |
|  |  | FUMOZ-R | 1 (11Dec) | **18** | 59 | 114 | 14.4 | 18.9 | 17.97 | 1.13 | 65.6 | 98.8 | 87.56 | 4.41 |
|  |  |  |  | **25** | 45 | 115 | 24 | 26.9 | 25.61 | 0.89 | 78.1 | 91.7 | 86.91 | 2.42 |
|  |  |  |  | **30** | 60 | 118 | 29.5 | 29.8 | 29.67 | 0.07 | 74.6 | 100 | 99.37 | 2.39 |
|  |  |  | 2 (16Feb)  +PBO | **18** | 29 | 101 | 17.2 | 19.0 | 18.74 | 0.24 | 81.1 | 100 | 97.73 | 2.87 |
|  |  |  |  | **18** | 26 | 101 |  |  |  |  |  |  |  |  |
|  |  |  |  | **25** | 25 | 101 | 21.8 | 26.3 | 24.44 | 0.91 | 74.5 | 83.0 | 79.93 | 1.83 |
|  |  |  |  | **25** | 28 | 104 |  |  |  |  |  |  |  |  |
|  |  |  |  | **30** | 26 | 104 | 30.3 | 30.8 | 30.49 | 0.16 | 57.5 | 88.9 | 81.90 | 4.78 |
|  |  |  |  | **30** | 23 | 100 |  |  |  |  |  |  |  |  |

|  |  |  |  |  | **n (# of females)** | | **Temperature (°C)** | | | | **RH (%)** | | | |
| --- | --- | --- | --- | --- | --- | --- | --- | --- | --- | --- | --- | --- | --- | --- |
| *Anopheles spp.* | Insecticide | Strain | Experimental replicate | Treatment | Control | Insecticide | Min | Max | Mean | SD | Min | Max | Mean | SD |
| *funestus* | Bendiocarb | FUMOZ | 1(17Apr) | **18** | 49 | 95 | 18.3 | 19.2 | 18.31 | 0.09 | 76.1 | 97.3 | 95.03 | 2.03 |
|  |  |  |  | **25** | 52 | 96 | 23.6 | 25.6 | 24.62 | 0.44 | 74.0 | 96.8 | 89.54 | 3.78 |
|  |  |  |  | **30** | 54 | 96 | 26.9 | 28.2 | 27.79 | 0.14 | 45.6 | 88.9 | 85.55 | 4.40 |
|  |  |  | 2(29Apr) | **18** | 40 | 100 | 18.3 | 21.8 | 18.41 | 0.04 | 69.4 | 94.6 | 92.70 | 2.07 |
|  |  |  |  | **25** | 49 | 100 | 24.1 | 26.6 | 25.16 | 0.55 | 74.5 | 95.9 | 87.43 | 3.69 |
|  |  |  |  | **30** | 51 | 99 | 29.8 | 30.6 | 30.39 | 0.48 | 41.8 | 92.5 | 87.15 | 5.78 |
|  |  | FUMOZ-R | 1(18Dec) | **18** | 48 | 98 | 18.3 | 22.4 | 18.42 | 0.37 | 55.2 | 92.1 | 88.20 | 5.98 |
|  |  |  |  | **25** | 49 | 96 | 23.4 | 28.3 | 26.28 | 1.63 | 66.3 | 85.2 | 74.45 | 4.64 |
|  |  |  |  | **30** | 38 | 96 | 29.3 | 29.8 | 29.40 | 0.11 | 58.2 | 98.4 | 93.44 | 4.86 |
|  |  |  | 2 (23Apr) | **18** | 51 | 101 | 18.3 | 19.1 | 18.37 | 0.06 | 76.6 | 94.3 | 93.06 | 1.47 |
|  |  |  |  | **25** | 48 | 98 | 23.9 | 25.2 | 24.70 | 0.31 | 81.0 | 98.3 | 89.96 | 3.40 |
|  |  |  |  | **30** | 51 | 91 | 30.4 | 30.9 | 30.64 | 0.12 | 83.5 | 93.0 | 90.91 | 2.21 |
|  |  |  | 3 (1May) | **18** | 40 | 111 | 18.3 | 20.1 | 18.46 | 0.13 | 53.6 | 90.6 | 88.97 | 2.96 |
|  |  |  |  | **25** | 50 | 98 | 23.3 | 25.6 | 24.56 | 0.58 | 77.9 | 97.2 | 88.42 | 3.52 |
|  |  |  |  | **30** | 44 | 106 | 29.2 | 30.8 | 30.59 | 0.11 | 30.1 | 87.3 | 84.40 | 5.67 |

^+^ 20 August measurements in 25°C conditions were every thirty minutes, rather than every five minutes

^*^ In experiments that included PBO treatments, the shaded “Control” column is the positive control, or the mosquitoes exposed to PBO-only, and the shaded “Insecticide” column are those mosquitoes exposed to deltamethrin+PBO.
